# Exploratory Research With a Health Consumer Group on Social Robot Use Among Older Adults: Qualitative Study

**DOI:** 10.2196/70462

**Published:** 2025-09-24

**Authors:** James Sadler, Aila Khan, Omar Mubin, Michael Lwin

**Affiliations:** 1 School of Business, Western Sydney University, 169 Macquarie Street, Parramatta, 2150, Australia, 61 497829851

**Keywords:** older adults, assistive technology, social robots, health care stakeholders, health consumer group, triple helix model of innovation, quality of life

## Abstract

**Background:**

There is an increased focus on involving members of the public in health research. These types of groups, such as “health consumer groups,” bring different expertise to inform the design of a research study. There is a growing general concern about older adults’ acceptance and use of technologies. This becomes critical when it involves health care services.

**Objective:**

To understand the use of social robots among older adults, it is prudent to gauge stakeholders’ perspectives on optimal research design. In line with the philosophy of the “triple helix model,” researchers sought the expertise and guidance of a health consumer group.

**Methods:**

Researchers recruited an expert health consumer group for this study. This included 5 participants from an 8-member panel. Semistructured interviews were conducted. Each interviewee was introduced to visual stimuli of assistive technologies, older adults, and social robots. Subsequently, they were asked for their perspectives on what they viewed and to provide guidance on how to best design upcoming research on these phenomena.

**Results:**

Key themes were derived from the interview transcripts with the health consumer group members. Findings include panel members’ advice and guidance on explaining the research aims to technology-averse older adults, approaching data collection from this demographic, and, finally, their perceptions of the appearance of social robots.

**Conclusions:**

The advice and guidance of this expert health consumer, in tandem with researchers and industry partners, substantially aid in advancing research efforts toward social robot use among technology-averse older adults in Australia. This research provides vital information, including how best to approach data collection about social robots from this demographic.

## Introduction       

### Health Consumer Groups

A health consumer group, also called the consumer council, is a platform that brings together community members to provide health care–related consumer views, opinions, and experiences to help guide research projects and to disseminate research findings among the community [1]. A “consumer” is a receiver or a potential receiver of health care services. However, other terms are also used to describe health care recipients, laypersons, users, service users, patients, and clients [2].

There is an increased focus on involving differing perspectives from the public domain in developing health-specific interventions [3]. This involvement of public representatives enables a broad range of differing ideas, perspectives, and resolutions to be brought forward [1]. Such public involvement is advocated for governing boards [4], social care [5], and consumer research [6]. It is recognized that the expertise of members of the public is unique and is different from possessing professional skills [7]. Consequently, this enables various stakeholders in the health care sphere to have their perspectives incorporated, enabling health care professionals to design and implement effective strategies [1]. While the use of “expert opinion” in health research is common [4], there is now a more significant push to increase the involvement of patients and the public in health research [8]. Public participation in health research refers to research conducted with or by the public rather than to, about, or for them [8]. Consumer and community participation in health care is well-recognized [9]. However, the case for public involvement in health research has, over the years, become more widespread [9].

Health consumer groups in the Australian health care networks are commonplace. Ordinarily, they comprise ex-health care professionals, retired professionals who worked adjacent to the health care sector, and stakeholders with a wealth of experience with the health care sector throughout their lives [1]. Subsequently, their perspectives are vital, as they offer an outside view of issues occurring within the health care sector, which may need to be seen or recognized by professionals [1].

### Rationale for Using Health Consumer Groups in Research

Social robot research among older adults in an Australian context is still emerging within the literature. Australia’s older adults are particularly hesitant and resistant to newer forms of technology, which pose expected barriers to the acceptance and use of social robots by Australia’s older adults in a clinical setting [10,11]. Enabling outside perspectives, such as those of members of a health consumer group, allows researchers to access valuable information regarding potential causes of these barriers. Theoretical, political, and ethical reasons are given in favor of public involvement with research. Theoretically, according to World Health Organization guidelines, there is a difference between disease and illness. A disease is a “physiological and clinical abnormality” [12]. On the other hand, an illness is a persistent sickness and is a “more subjective concept related to personal experience of a disease” [12]. Research studies require the expertise of professionals and input from “experts” who may have witnessed and experienced living through a medical condition.

The political argument for involving the public members in health research is based on the premise that, as citizens and taxpayers, individuals who use health care services have the right to influence research funded through public monies [13]. Finally, the traditional model of a passive patient and an active doctor is no longer valid. Similar to the ethical obligation of health to involve patients in service delivery [2], there is a growing call for community input in research conducted within those communities.

### Roles Played by Members of Health Consumer Groups

Members of health consumer groups play a critical role in ensuring clinically relevant research is being undertaken. Health group members are essential in identifying priority areas for research. They can identify research gaps from a layperson’s perspective [13]. First, consumer participation helps determine the type of information communities need [13]. Second, they can help with the design and development of innovations. Next, they can help recruit relevant participants for research studies [8]. Finally, members of a health consumer group can ensure that the information produced is in a format and language that is easily comprehensible [13].

### Limitations of Health Consumer Groups

There are challenges in using health consumer groups for exploratory research. First, as a preselected group, the perspectives of the consumer group members may not be representative of opinions held by the entire demographic or community [14]. Second, the size of the consumer group plays a crucial role in the depth of the discussion conducted in qualitative research [14]. If a consumer group is too large and the resources of researchers are spread too thin, the engagement can become disorganized, difficult to manage, and lead to inadequate data collection [15]. If the consumer group is too small, the scope of information could be further limited [15]. Finally, there is a chance of misunderstanding some of the technical information and jargon [15]. Not all health consumer group members are health care professionals. This may create knowledge deficits or misunderstandings and can lead to misconceptions about the research, interventions, policies, or practices discussed within the interview processes [14].

### Triple Helix Model

**Figure 1.**
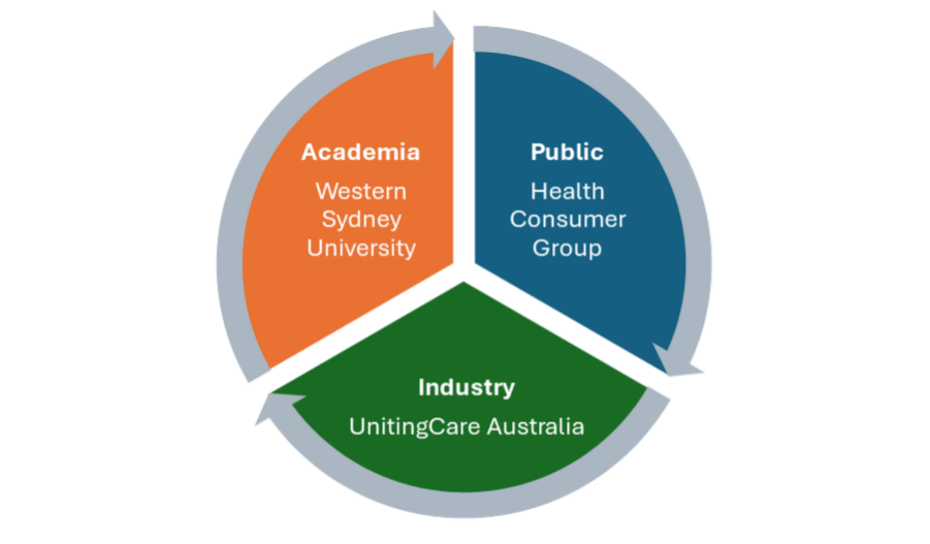
The triple helix model (as applied in this study).

The triple helix model (Figure 1) inspired the use of a health consumer group for this study. First proposed by Etzkowitz and Leydesdorff [16], the “triple helix model” (Figure 1) initially referred to the interactions among higher education, government, and industry to encourage economic, social, and cultural development in a region. Each component of the helix model plays a commensurate role in fostering economic, social, and cultural development [17]. For example, higher education focuses on research and the dissemination of innovation, industry on the production of goods and services, and the government or the public on serving the interests of people, including legislative and regulatory mechanisms that encourage and foster the diffusion of innovation into a population [17].

The triple helix model has mainly been used to improve the innovation process [16]. However, this study uses an “end-user enriched triple helix*”* approach [18], which considers the end-users’ perspective. Innovations do not occur spontaneously; in fact, understanding the user’s perceptions and interacting with them is critical to the success of a technological innovation [19]. The lack of input from users may lead to missed opportunities. The use of the triple helix model is based on the premise that involving the end users and adopting a participatory approach would lead to a more effective innovation development process. As older adults are the logical end users [18] of such technologies as social robots in the aged care sector, the triple helix model provides an essential apparatus to include their perspectives in the research process while incorporating academic and industry interests [17].

### Social Robots and Older Adults

**Figure 2.**
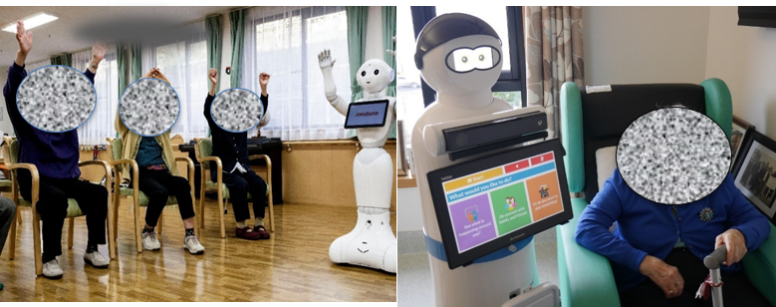
(A) Pepper leading group physio. (B) Mario along with a patient with Alzheimer disease (credit: Getty Images).

Assistive technology use among older adults is commonplace. Assistive technologies are ordinarily used to improve the quality of life of older adults by overcoming a physical or cognitive deficit [20]. Newer forms of assistive technology, such as social robots, have been deployed among older adults in a range of contexts with demonstrable effectiveness (Figure 2A). Social robots are machines equipped with a degree of artificial intelligence and human-like mechanics that can mimic or replicate a human interaction with a user [20]. These interactions include companionship, gameplay, touch, eye movement, handshaking, hugging, greetings, verbal interactions, and recognizing cues (Figure 2B). In the aged care sphere, where the number of older adults is increasing, and the number of care staff is declining, social robots can provide valuable supplements to care practices, social interactions, connectedness, and subsequent improvements in the quality of life for older adults (Figure 2B) [21,22].

Primarily, research on social robots among older adults has focused on mimicking social interactions and providing companionship (Figure 2B) [23,24]. As demonstrated by Khosla et al [25], interactions between social robots and older adults in care were associated with significant improvements in observed states of well-being among residents. This is reinforced by Ge and Schleimer [26], whose analysis of older adults’ use and acceptance of social robots demonstrated a marked improvement in general well-being. The benefits of social robots to older adults in care transcend social interactions and companionship. Consistent with Fasola and Mataric [27] and Kyong et al [28], deploying a socially assistive robot to assist in exercise routines has been shown to provide positive motivation and outcomes for older adults in care settings. Socially assistive robots can also motivate older adults to engage in physical activities (Figure 2A). According to Macis et al [29], the deployment of a socially assistive robot into a residential aged care facility motivated residents to try and maintain a daily stretching and tai chi routine. Pharmacological disbursement is another area where socially assistive robots have been deployed effectively. According to Smarr et al [30], reminders to take medications and management systems for pharmacology by socially assistive robots in care facilities have produced positive outcomes and a greater level of compliance.

Studies show that the use of social robots can improve the quality of life; however, acceptance of the technology is a key barrier in Australia [31]. To better explore and understand this phenomenon, researchers contend that it is important to seek broader contextual information from differing players in the health care sphere, such as assessing the acceptance of the technology by a health consumer group. This would aid in overcoming these barriers in Australia. In addition, Australia has an aging population, and it is expected that the proportion of Australians aged 65 and older will represent 21%‐23% of the population in 2066. As mentioned above, with a major shortage of health care workers in Australia, the social robots in aged care present an opportunity to fulfill this gap. Thus, understanding the adoption of social robots in aged care in Australia has become a national priority.

### Research Objectives

Social robot research among older adults in an Australian context is still in its infancy. Research using a health consumer group in designing an academic study with older adults and social robots is limited. Before testing social robots as an intervention among older adults in a clinical aged care setting, it is prudent to gauge the perceptions of an expert health consumer group. Subsequently, the objective of this research is to apply the triple helix model of innovation to seek guidance from a health consumer group to aid research efforts in the adoption of social robots among older adults.

## Methods

### Participant Recruitment

UnitingCare Australia recommended the research team contact the Sydney Partnership for Health, Education, Research and Enterprise for advice on this study. An element of the Sydney Partnership for Health, Education, Research and Enterprise is the Age and Aging Clinical Academic Group (AAA CAG), which acts as an expert health consumer panel related to aged care [1]. The work of the AAA CAG is to guide and inform research, policy, practice, and support assistive technology use to further healthy aging and independent living for Australia’s aged population [1]. The AAA CAG consists of 8 members, many of whom have worked within, adjacent to, or have extensive experience in the Australian health care sector. People who worked in AAA and CAG formed the population of the study. To reduce bias, each person was invited to participate in the study. Therefore, the participants were selected randomly from the population.     

### Participant Information

Overall, 5 members of AAA CAG were interviewed, of whom 3 were deselected due to lack of interest, age, and time constraints. Of the 5 interviewed members, 3 were males and 2 were females between the ages of 66 and 73 years. Overall, 2 were from non–English-speaking backgrounds (non-ESB) and 3 were from English-speaking backgrounds (ESB; Table 1.

**Table 1.** Participant demographic information.

Participant number	Age (years)	Sex	Language background
P1	73	Male	Non–English speaking
P2	66	Male	English speaking
P3	73	Female	English speaking
P4	72	Female	Non–English speaking
P5	66	Male	English speaking

### Participant Interviews

Semistructured interviews were conducted with each member via Zoom (Zoom Communications, Inc.). Participants were asked background questions from a preselected pool about their perceptions and experiences with assistive technologies. Visual stimuli, including images and short video clips about social robots and older adults, were used to give the participants an insight into this novel technology. Examples included images of a social robot leading a seated physiotherapy routine to older residents in a care facility. Similarly, a BBC YouTube clip of an independently living older man interacting with PEPPER, a social robot, was also used in the interview process. This was designed to mirror past studies that have also used videos and visual stimuli to demonstrate the capability of robots [32,33]. Finally, researchers asked participants for their opinions, feelings, and perceptions regarding the use of social robots in a research project with older adults. Each interview lasted approximately 45 minutes, and audio recordings were taken using Zoom. Professional transcriptions of each recording were generated using Notta AI software (Notta Inc.), and each participant was deidentified and provided with a participant number (P1-P5). Manual content analysis was conducted on each transcription. Transcripts were read and reread manually to ensure maximum retention.

### Data Analysis

The initial content analysis was inductive, following the guidelines of Braun and Clarke [34,35]. This involved familiarization with the data by reading and rereading the interview transcripts. During this process, the researchers identified and reviewed initial codes, which were later grouped into themes. The themes were subsequently confirmed through meetings with the research team and deductively grouped using the protocols developed by the Australian Clinical Trials Alliance. Three key themes emerged across the 5 interviews with members of the health consumer panel.

### Ethical Considerations

Ethics approval was sought from the Western Sydney Human Research Ethics Committee, which approved application H15666 to seek information from the AAA CAG. As the study involved an expert consumer panel, who is involved with aged care in an ancillary capacity, its ethics classification was a low-negligible risk. The AAA CAG meets monthly to discuss various research projects, technologies, interventions, policies, and practices currently within the health care sector. As part of the recruitment process, the authors of this paper were asked to present their research study via a Zoom meeting to all AAA CAG panel members. The chair of AAA CAG permitted the research team to contact each interested member for interviews by providing each member’s email address. Interviews, rather than a focus group discussion, were preferred. Interviews ensured that all members were free to voice their opinions without hesitation. Each participant met the inclusion criteria: a member of the AAA CAG, above 65 years of age, willingness and availability to participate in the study, and some experience with aged care consumer products. After the preselection phase, researchers distributed participant information sheets, consent forms, and other informational materials to members who indicated interest in participating. Informed consent was obtained prior to conducting interviews. All participants were deidentified and any identifiable information was obscured within this research and held by researchers only. Gift cards in the amount of $50 were distributed to each participant for their participation in this project.

## Results

### Overview

This section outlines the main themes that emerged as the researchers analyzed the transcripts. In line with previous research on engaging consumers and communities [36], we focused on identifying topics that provided guidelines for researchers on how to use social robots with older adults. Participants’ quotes are presented with the following information: participant identification number, gender, age, and language background (ESB or non-ESB).

### Communicating the Research Aim to Tech-Averse Participants

Researchers may find communicating the interview’s research aims to the participants easy and straightforward. However, it can be a challenge with some population segments. Older adults could be skeptical about new technologies, including social robots. Not surprisingly, some may hold opposing views even about standard assistive technologies.

*… my mother-in-law - an elderly lady-had to be convinced to get a walking stick and get it fitted properly as well. She said she didn’t need it, but she does*.

[P3, Female, 73 years, ESB]

Hesitation to use tools or equipment by older adults appears to be shared across different backgrounds and demographics:

*… it’s nothing to do with education. It’s to do with a mindset* [of this age group].

[P4, Female, 72 years, non-ESB]

One research participant was particularly vocal about the need to explain the research aims to older adults:

*… the biggest thing that I can see is to be able to communicate, talk to them [participants].*

[P1, Male, 73 years, non-ESB]

The same participant offered advice, stating:

*Initially, they might [resist], but when they can see the benefits of it, I don’t think there will be any resistance [to the research project*].

Panel members of this consumer health group reiterated that undertaking human-robot interaction research is meaningful if older adults are convinced about its benefits. As mentioned by a non–English-speaking panel member:

*Initially, they might* [resist], *but when they can see the benefits of it, I don’t think there will be any resistance*...

[P1, Male, 73 years, non-ESB].

A suggestion on how to communicate the research aims of a social robot project to older adults highlighted the role of family members:

*If there was a family member or carer involved and they also were accepting and recommending [involvement in the research project], I think yes... that would certainly get through the first barrier*.

[P5, Male, 66 years, ESB]

### Data Collection Methods With Older Adults

The consumer health panel members advised researchers on collecting data from older adults. The participants’ overarching opinions were “direct” questions and “face-to-face” interviews. The research team also received advice on involving “health professionals” and “family members” during the data collection phase. One panel member (P1, male, 73 years, ESB) warned the research team against using videos to communicate the idea of a social robot. Despite older adults’ generally known aversion to technology, this English-speaking male participant strongly advocated for a direct interaction of the robot with older research participants.

### Appearance of the Social Robot

The research team also sought advice on the robot’s appearance. Two members of the consumer panel had previously been exposed to humanoids:

*...In Paris, in 2017, ... this little white machine came up and was talking to us in English.*

[P3, Female, 73 years, ESB]

Another participant revealed the introduction of a baby seal, Paro, at his previous workplace. These panel members did not find the social robot “scary.” Such participants quickly commented that this may not be the case with the average older adult who would interact with the humanoid for the first time:

*Look..., it goes back to that idea of dystopian...it’s sort of big brotherish*.

[P5, Male, 66 years, ESB]

Another panel member (P4, female, 72 years, non-ESB) stated that while the robot’s image was “acceptable”… “the eyes could be more friendly.” This participant also hesitated about the robot’s look:

*The expression,..., the expression, yeah*...

[P4, Female, 72 years, non-ESB]

Interestingly, another female participant (P3, 73 years, ESB) appreciated the “feminine form” of the robot and that it was “not confronting with the height and size.”

## Discussion

### Principal Findings

In this section, we highlight 3 key themes identified from the data analysis. We also highlight the managerial implications of the results for each theme. The primary aim of this paper was to seek advice and guidelines from members of a health consumer group regarding human-robot interaction research with older adults. Such advice becomes even more critical when the consensus is that a participant group is vulnerable [37]. There is a general concern about older adults’ vulnerability to technology, especially regarding their security and privacy [38].

### Key Theme 1: Communicating the Research Aim to Tech-Averse Participants

The first theme that emerged from our analysis was the importance of explaining the aims of a research project to an anxious participant group. Previous researchers [39] have already acknowledged that conducting research with older adults presents challenges; however, the most identified challenges center around obtaining consent and ensuring privacy during interviews. The discussion with the health consumer group revealed that presenting the research topic to tech-averse participants, such as older adults, could hinder gaining cooperation for interviews and discussions [40]. The results demonstrate that demographic variables have little role to play in older adults’ attitudes toward technology. Researchers working on tech-related topics should involve family members, peers, or other familiar clinicians to ensure smooth participation in the research project. Furthermore, identifying an opinion leader within the group and encouraging them to use technology will have a positive impact on the group’s adoption of the technology. This snowball effect will be more powerful than trying to convince everyone to adopt the technology. In addition, to encourage tech-averse participants to adopt the technology, consistent messaging about its benefits is required. For example, “the robot can help with my exercise routine” and “the robot exercise routine is entertaining and fun.” Highlighting the health and entertainment benefits is more likely to reduce resistance to technology. Furthermore, it is also important to change the mindset; for example, a 30-minute robot tai chi exercise as part of their daily routine could help change their mindset. Robots delivering these exercises daily can help older individuals change their daily behavior and change their mindset toward regular exercise.

### Key Theme 2: Data Collection Methods With Older Adults

Another commonly discussed topic with the health consumer group focused on data collection methods. Despite increasing evidence of older adults’ comfort in using the internet [41], our panel of experts felt that the best way to collect data was face-to-face, allowing older adults to interact directly with a social robot. While it may not be feasible to “house” the robot for extended periods at a facility [42], the findings suggest short-term interactions in a safe environment would be an ideal way to introduce older adults to humanoids. For example, short 30-minute robot-driven exercises daily would work better than 2‐3 hours of activity. The findings also indicate that effective communication between the robot and the individual is critical in the adoption of the robot. Older individuals would find it difficult to communicate with the robot if it were at the house for only 1‐2 days. Thus, having the robot for several weeks or months in the house would help older individuals learn to communicate with the robot more effectively. This would also help change the “mindset” toward being more active and exercising.

### Key Theme 3: Appearance of the Social Robot

Finally, the health consumer group provided mixed feedback on the robot’s appearance and features. Recent studies [43] have found that older adults label the robot as “friendly.” However, it could be that some of these studies used smaller types of robots in their projects. Other researchers [44] have reported older adults’ dislike for a machine that tries to look like a human—a fake human. Our results demonstrate the need for further research in this area. It is necessary to consider users’ needs by investigating them in greater detail. At this stage, the results show conflicting arguments from the health consumer group. For example, some preferred robots with eyes that were more friendly. However, they did not like the expressions of the robot. Other participants highlighted that the feminine features of the robot made it look less confrontational. While these findings are limited to a few participants, it is clear that the personalization and customization of robot features are necessary to enhance adoption. It was interesting to find that the adoption of the robot depends on the individual’s preferences for the robot’s features. However, academic research in this area is limited, and future studies should investigate the personalization and the personification of robots.

### Limitations and Future Studies

Several essential caveats deserve comment. First, this research study was conducted with an 8-member health consumer group, resulting in a small sample size for interviews. Next, our study considered only one type of social robot—Pepper. It is quite possible that a different robot type may have resulted in different opinions by the participants. Finally, it is important to consider the presence of bias in participant responses due to their values and attitudes toward robots. The industry partner (UnitingCare Research) recommended future research to focus on the health consumer group. This would provide a standard unit of analysis in future studies, and it provides researchers with a standard research process to compare against. One of the key learnings from the study is that when you are recruiting participants for the health consumer group, it is important to vet the participants. Future studies should develop a vetting process for the health consumer group. This would help future studies have a standardized selection process and help researchers compare the results more accurately. Furthermore, the study was limited to the qualitative methodology, and future studies should validate the findings using empirical modeling.

As this research is the foundation for future studies, including social robots and older adults, the results highlight how best to approach studying this phenomenon. Future studies should consider these recommendations to effectively design social robot interventions among older adults in the Australian aged care context. However, the findings are limited to the Australian aged care context, and future researchers should explore different cultural backgrounds. For example, in the Middle East, religion is at the core of their livelihood, and therefore, the way individuals interact with the robot may differ substantially from that of older Australian adults. The Japanese are more likely to adopt the robot; thus, future researchers may compare whether culture impacts the adoption process. Comparisons between these contexts are important to understand the factors driving robot adoption. These findings could be used to advise future studies in aged care.

### Conclusions

Health consumer groups are vital health care stakeholders, whose experiences, perspectives, concerns, and recommendations form the basis for designing robust interventions to improve the quality of life for specific demographics through products and services. After being pretested among an expert health care consumer panel by the authors, the findings of this study should provide confidence to another element of the triple helix model: industry. However, as mentioned in the limitations of this study, social robot research among older adults in Australia is still emerging. This study represents nascent exploratory research that requires further testing. The authors, in conjunction with industry partners, recommend field testing of social robots among older adults to assess their acceptance and use, as they are the intended end users of the technology.

## Abbreviations

CAG: Age and Aging Clinical Academic Group
ESB: English-speaking background
non-ESB: non–English-speaking background
